# Supplementary material for: Genomic regions controlling shape variation in the first upper molar of the house mouse
Source: eLife. 2017 Nov 1;6:e29510. doi: 10.7554/eLife.29510 (PMC5679752; doi:10.7554/eLife.29510)
Supplement: Supplementary file 1. — A Hotelling T2 test was performed to evaluate the difference in mean shape between mutant and wildtype groups; p-value, test statistic, and sample size (N) are shown. The Procrustes distances between mutant and wild type mean shapes are also indicated. *The comparison between heterozygous and homozygous mice for the Mitfmi-vga9 mutation is also shown. (B) Missense variants found in the gene Mitf of wild mice. Nine populations of wild mice were screened for SNPs causing coding changes in Mitf: Mus musculus musculus from Kazakhstan, Check Republic, and Afganistan; M. m. domesticus from Iran, Heligoland, France, and Germany; Mus castaneus; and Mus spretus. Data available in the UCSC browser → MyData - > Public Sessions - > wildmouse (Harr et al., 2016). In addition, eight hybrids between M.m. musculus and M.m. domesticus from the German hybrid zone (Turner, Tautz and Harr unpublished data), the same population used in this study, were also screened for coding changes. Reference and variant alleles are shown. nVar = number of chromosomes with the variant allele, n = number of chromosomes per population. [file elife-29510-supp1.docx]

**Supplementary file 1A.** Shape comparison between *Mitf* mutants and wild-type B6 mice. A Hotelling T^2^ test was performed to evaluate the difference in mean shape between mutant and wildtype groups; p-value, test statistic, and sample size (N) are shown. The Procrustes distances between mutant and wild type mean shapes are also indicated. *The comparison between heterozygous and homozygous mice for the *Mitf^mi-vga9^* mutation is also shown.

|  | *Mitf^mi-enu122^/ Mitf^mi-enu122^* | *Mitf^Mi-wh^*/*Mitf^mi^* | *Mitf^mi-vga9^*/+ | *Mitf^Mi-wh^*/*Mitf^Mi-wh^* | *Mitf^mi-vga9^*/ *Mitf^mi-vga9^* | *Mitf^mi-vga9^* het vs hom* |
| --- | --- | --- | --- | --- | --- | --- |
| Hotelling T2 | p=0.0006 T^2^(2,12)=20  N=15 | p=0.011 T^2^(2,7)=9  N=10 | p=0.0042 T^2^(2,7)=19  N=10 | p=0.0008 T^2^(2,6)=49  N=9 | p=0.0006 T^2^(2,7)=56  N=10 | p= 0.0076  T^2^(2,7)=13  N=10 |
| Procrustes distance | 0.0239 | 0.0253 | 0.0300 | 0.0372 | 0.0466 | 0.0243 |

**Supplementary file 1B.** Missense variants found in the gene *Mitf* of wild mice. 9 populations of wild mice were screened for SNPs causing coding changes in Mitf: *Mus musculus musculus* from Kazakhstan, Check Republic, and Afganistan; *M. m. domesticus* from Iran, Heligoland, France, and Germany; *Mus castaneus;* and *Mus spretus.* Data available in the UCSC browser 🡪 MyData -> Public Sessions -> wildmouse (Harr *et al.* 2016). In addition, 8 hybrids between *M.m.musculus* and *M.m.domesticus* from the German hybrid zone (Turner, Tautz & Harr unpublished data), the same population used in this study, were also screened for coding changes. Reference and variant alleles are shown. nVar = number of chromosomes with the variant allele, n = number of chromosomes per population.

| **Location (mm10)** | **Ref/Var** | **Feature type** | **nVar/n** | **Amino acid change** | **Codon change** | **Species - population** |
| --- | --- | --- | --- | --- | --- | --- |
| chr6:97941094 | G/A | Transcript | 1/16 | S/N | aGc/aAc | M.m.musculus - Kazakhstan |
| chr6:97941156 | G/C | Transcript | 1/20 | E/Q | Gag/Cag | M.castaneus |
| chr6:97941187 | T/A | Transcript | 2/20 | L/Q | cTg/cAg | M.castaneus |
| chr6:97993296 | G/A | Transcript | 1/20 | S/N | aGc/aAc | M.castaneus |
| chr6:98017825 | G/A | Transcript | 6/16 | G/S | Ggt/Agt | M.spretus |
| chr6:98017984 | A/C | Transcript | 6/16 | M/L | Atg/Ctg | M.spretus |
| chr6:98018168 | C/T | Transcript | 6/16 | A/V | gCg/gTg | M.m.domesticus - Germany |
| chr6:97994441 | C/T | Transcript | 3/16 | H/Y | Cac/Tac | Hybrid mice - Bavaria |

Harr, B., Karakoc, E., et al. (2016). "Genomic resources for wild populations of the house mouse, Mus musculus and its close relative Mus spretus." Sci Data 3: 160075.
